# Cell surface GRP78 regulates BACE2 via lysosome-dependent manner to maintain mesenchymal phenotype of glioma stem cells

**DOI:** 10.1186/s13046-020-01807-4

**Published:** 2021-01-07

**Authors:** Zihang Chen, Huizhi Wang, Zongpu Zhang, Jianye Xu, Yanhua Qi, Hao Xue, Zijie Gao, Rongrong Zhao, Shaobo Wang, Shouji Zhang, Wei Qiu, Xing Guo, Gang Li

**Affiliations:** 1 Department of Neurosurgery, Qilu Hospital, Cheeloo College of Medicine and Institute of Brain and Brain-Inspired Science, Shandong University, Jinan, Shandong China; 2 Shandong Key Laboratory of Brain Function Remodeling, Jinan, Shandong China; 3 Department of Neurosurgery, Qilu Hospital of Shandong University, 107 Wenhua Western Road, Jinan, 250012 Shandong China

**Keywords:** GRP78, Glioma stem cell, BACE2, Radiation, Mesenchymal subtype

## Abstract

**Background:**

Glioma stem cells (GSCs) are considered the initial cells of gliomas, contributing to therapeutic resistance. Patient-derived GSCs well recapitulate the heterogeneity of their parent glioma tissues, which can be classified into different subtypes. Likewise, previous works identified GSCs as two distinct subtypes, mesenchymal (MES) and proneural (PN) subtypes, and with general recognition, the MES subtype is considered a more malignant phenotype characterized by high invasion and radioresistance. Therefore, understanding the mechanisms involved in the MES phenotype is necessary for glioblastoma treatment.

**Methods:**

Data for bioinformatic analysis were obtained from The Cancer Genome Atlas (TCGA) and The Gene Expression Omnibus (GEO) database. An antibody was used to block cell surface glucose-regulated protein 78 (csGRP78). Apoptosis and cell cycle analyses were performed to evaluate radiation damage. Immunofluorescence staining was applied to assess protein expression and distribution. Mass spectrometry combined with bioinformatic analysis was used to screen downstream molecules. Intracranial GSC-derived xenografts were established for in vivo experiments.

**Results:**

Total GRP78 expression was associated with MES GSC stemness, and csGRP78 was highly expressed in MES GSCs. Targeting csGRP78 suppressed the self-renewal and radioresistance of MES GSCs in vitro and in vivo, accompanied by downregulation of the STAT3, NF-κB and C/EBPβ pathways. Mass spectrometry revealed the potential downstream β-site APP-cleaving enzyme 2 (BACE2), which was regulated by csGRP78 via lysosomal degradation. Knockdown of BACE2 inactivated NF-κB and C/EBPβ and significantly suppressed the tumorigenesis and radioresistance of MES GSCs in vitro and in vivo.

**Conclusions:**

Cell surface GRP78 was preferentially expressed in MES GSCs and played a pivotal role in MES phenotype maintenance. Thus, blocking csGRP78 in MES GSCs with a high-specificity antibody might be a promising novel therapeutic strategy.

**Supplementary Information:**

The online version contains supplementary material available at 10.1186/s13046-020-01807-4.

## Background

Glioblastoma multiforme (GBM) is the most prevalent and aggressive malignant tumor of the central nervous system, with uniformly poor survival [1]. Previous studies have reported that GBMs and glioma stem cells (GSCs) exhibit apparent cellular heterogeneity [2, 3]. GSCs, characterized by unlimited self-renewal and tumorigenic capacities, are considered the initiating cells of GBMs [4, 5]. Thus, GSCs play a crucial role in the malignant progression of GBMs, and targeting GSCs has been shown to be a therapeutic regimen for improving patient survival. Based on gene expression signatures, GBMs can be classified into three major subtypes: proneural (PN), classical (CL) and mesenchymal (MES). Each subtype shows high expression of its characteristic markers, such as SOX2 and OLIG2 for the PN subtype and CD44 and YKL40 for the MES subtype [2]. In addition, patients with PN GBMs exhibit more favorable outcomes and higher radiotherapeutic sensitivity than those with MES GBMs [6, 7]. Similarly, GSCs were mainly classified into PN and MES subtypes. As the MES subtype of GSCs is often associated with higher radioresistance and is more common in recurrent GBMs, it is indispensable for improving the therapeutic efficacy to uncover the mechanisms involved in MES characteristic acquisition and maintenance. To date, four major transcription factors, STAT3, C/EBPβ, TAZ, and NF-κB, are well defined to play an essential role in regulating malignant progression, especially mesenchymal differentiation and maintenance of GSCs [7, 8].

Glucose-regulated protein 78 (GRP78) has been functionally defined as a stress-inducible molecule that facilitates aggressive growth and radioresistance in various tumors, including GBM [9, 10]. In addition to its cytosolic distribution, GRP78 can relocalize to the cell surface as a receptor by which oncogenic pathways are activated to regulate proliferation, apoptosis, and motility [11, 12]. Previous studies have demonstrated that csGRP78 is involved in the malignant behaviors of high-grade glioma and that blocking csGRP78 suppresses the radioresistance of GBM [13, 14]. More importantly, csGRP78 regulates the biological behaviors of tumor stem cells [15, 16]. Suggested by these findings, it is possible to find the same translocation of GRP78 to the cell surface in GSCs and further explore what role it plays in tumor initiation and therapeutic resistance. To date, the expression and function of csGRP78 in certain subtypes of GSCs have been less well explored.

In this study, we aimed to explore the effects of GRP78 on GSCs with an identified subtype, as well as its downstream and specific mechanism, shedding light on the potential use of this classic molecule as a therapeutic target in GBM.

## Materials and methods

### Cell lines and reagents

All patient-derived GSC cell lines and neural progenitor cell (NPC) were kindly donated by Dr. Krishna P.L. Bhat (The University of Texas, M.D. Anderson Cancer Center, Houston, TX). GSC lines (PN: GSC11, GSC8–11; MES: GSC20, GSC267) were established and widely applied in previous studies [7, 8, 17], whose subtype had already been identified by metagene score for PN or MES subtype based on Philips and Verhaak gene set respectively [2, 6]. GSC lines and NPC were cultured in DMEM/F12 (10565018; Gibco, USA) supplemented with 2% B-27 no serum supplement (17,504,044; Gibco, USA), 20 ng/mL human recombinant EGF (236-EG; R&D Systems, USA) and 20 ng/mL human recombinant bFGF (233-FB; R&D Systems), being incubated at 37 °C in 5% CO_2_. Accutase solution (A6964; Sigma-Aldrich, USA) was used to digest the tumor spheres. All cells had been proved free of mycoplasma contamination before the study beginning.

Poly-L-ornithine solution (P4957; Sigma-Aldrich) and Laminin (L4544; Sigma-Aldrich) were used to pre-coat cell culture plates. Chloroquine (C6628; Sigma-Aldrich) was solved to 1 mM with ddH_2_O, and MG-132 (S2619; Selleck, USA) was stored on 5 mM with DMSO (D5879; Sigma-Aldrich). GRP78 polyclonal antibody (ADI-SPA-826; Enzolifesciences, USA) was applied to blockade cell surface GRP78 (described as anti-GRP78), setting isotype control with rabbit IgG isotype control (3900; Cell Signaling Technology, USA).

### Western blotting and antibodies

Cell deposit was washed by cold PBS and lysed with RIPA containing 1% protease and phosphate inhibitor cocktail (P8340; Sigma-Aldrich, USA). After the SDS-PAGE gel electrophoresis, transferring protein to the polyvinylidene fluoride (PVDF) membrane. Then primary antibodies were used to incubate strips at 4 °C overnight and probed by secondary antibodies. Following primary antibodies were used: β-actin (8480; proteintech, USA; 1:10000), GAPDH (ab8245; Abcam, UK; 1:1000), GRP78(3177; Cell Signaling Technology, USA; 1:1000), cleaved-PARP (32,563; Cell Signaling Technology; 1:1000), γ-H2AX (9718; Cell Signaling Technology; 1:1000), CD44 (3570; Cell Signaling Technology; 1:1000), YKL40 (ab77528; Abcam; 1:1000), SOX2 (3579; Cell Signaling Technology; 1:1000), Olig2 (ab109186; Abcam; 1:1000), phospho-NF-κB p65 (3031; Cell Signaling Technology; 1:1000), NF-κB p65 (8242; Cell Signaling Technology; 1:1000), phospho-STAT3 (9145; Cell Signaling Technology; 1:1000), STAT3 (4904; Cell Signaling Technology; 1:1000), C/EBPβ (ab32358; Abcam;1:1000), BACE2 (ab5670; Abcam; 1:500 And sc-271,212; Santa Cruz Biotechnology, USA; 1:250), DYKDDDDK Tag (8146; Cell Signaling Technology; 1:1000), Plasma Membrane Fraction Western blotting Cocktail (ab139413; Abcam; 1:200).

### Reverse transcription and qRT-PCR

Total RNA of GSCs was extracted by using TRIzol, according to the manufacturers’ protocol. Reversed transcription and qRT-PCR were performed as previously mentioned [18]. The sequences of primers were detailed in the Additional file 1: Table S1.

### Neurosphere formation assay

GSC tumor spheres were dispersed by accutase solution and implanted into 6-well plates at 1000 cells per well. After 1 to 2 weeks incubation with GSC culture medium, neurosphere formation was compared between groups using a Leica microscope to acquire images and measure sphere diameters for quantification analysis.

### Extreme limiting dilution assay (ELDA)

GSCs were implanted in ultralow-attachment 96-well plates in a density gradient of 0, 2, 4, 8, 16, 32 and 64 cells per well with 10 replicates. Seven days after implantation, the number of wells with successful formation of tumorspheres was counted. Data were analyzed by ELDA software(http://bioinf.wehi.edu.au/software/elda/) [19].

### Plasmid construction and transfection

To overexpress GRP78 in GSCs, we constructed the WT full-length FLAG-GRP78 plasmid, which expressed GRP78 that could relocalize to the cell surface after transfection, according to previous studies [20]. The detailed sequence was kindly supplied by Amy Shiu Lee. GeneChem (Shanghai, China) was responsible for constructing the plasmid Utr-NM_005347(ins_flag) using GV219 as the vector and for providing the control plasmid. We used Lipofectamine 3000 to transfect GSCs with the FLAG-GRP78 plasmid following the manufacturer’s protocol. Forty-eight hours after transfection, the protein expression of csGRP78 (with C-terminus FLAG tag) was assessed by immunofluorescence and western blotting.

### RNA interfering

The transient knockdown of GRP78 and BACE2 was achieved using small interfering RNA (siRNA), following the recommended protocol of lipofectamine 3000 (L3000015; Invitrogen, USA), and experiments were performed 72 h after transfection. Two independent sequences were used for western blotting. The persistent knockdown of GRP78 and BACE2 was performed by Lentivirus (GenChem, China) expressing shRNA (shGRP78 or shBACE2). The procedure was accord with the manufacturers’ protocol for suspended and stem cells. GSC culture medium containing 2 μg/mL puromycin was prepared for the subculture of transfected GSCs. The sequences of all RNA interfering were detailed in the Additional file 1: Table S1.

### Assessment of apoptosis by TUNEL

We applied one step TUNEL apoptosis assay kit (C1090; Beyotime) to detect the apoptosis of GSCs in each group after treatment, in accordance with the manufacturer’s protocol. In brief, GSCs attached to culture plates were rinsed with PBS, fixed with 4% paraformaldehyde, and permeabilized with PBS containing 0.5% Triton X. Then, the detection solution was prepared for staining according to the instructions. DAPI solution was required for nuclear staining (C1006; Beyotime). The proportion of apoptotic cells (red fluorescence), was observed and imaged using Lecia microscope.

### Assessment of apoptosis by Annexin V and propidium iodide (PI) staining

We applied FITC-Annexin V apoptosis detection kit (556,547; BD Biosciences, USA) to detect proportion of apoptosis, according to the manufacturer’s protocol. A FITC-conjugated anti-Annexin V antibody was replaced with an APC-conjugated anti-Annexin V antibody (550,474; BD Biosciences) for GFP-positive GSC cells. Sample collection and analysis were performed using a BD Accuri C6 flow cytometer (BD Biosciences).

### Cell cycle analysis

After treatment, GSC neurospheres were collected by centrifugation and dissociated with accutase solution. Then, the cells were washed with PBS and resuspended in cold 70% ethanol for fixation overnight at 4 °C. After fixation, the cell pellets were collected and washed with PBS to remove ethanol. Then cells in every sample were resuspended with 500 μl of PI/RNase Staining Buffer (550,825; BD Biosciences) and incubated for 15 min at room temperature. The cell cycle of GSCs was analyzed by a BD Accuri C6 flow cytometer.

### Immunofluorescence staining

Suspended GSCs were attached to μ-slide 8-well plates (80,826; ibidi, Germany) and treated with reagents before staining. The cells were washed gently with PBS and fixed with 4% paraformaldehyde at room temperature for 15 min. If necessary, treatment with 0.1% Triton X-100 in PBS at room temperature for 2 min was applied to permeabilize the cells. After rinsing with PBS three times, the cells were blocked with 5% goat serum (16,210,064; Gibco, USA) in PBS for 30 min at room temperature. Then, the cells were incubated with diluted primary antibodies in PBS at 4 °C overnight. The following primary antibodies were used: mouse GRP78 Monoclonal Antibody C38 (14–9768-37; eBioscience, USA; 1:200), rabbit anti-BACE2 (ab5670; Abcam; 1:400), mouse anti-BACE2 (sc-271,212; Santa Cruz Biotechnology; 1:50), mouse anti-DYKDDDDK Tag (8146; binds to the same epitope as Sigma’s anti-FLAG antibody; Cell Signaling Technology; 1:500), and rabbit anti-LAMP1 (9091; Cell Signaling Technology; 1:200). Rabbit IgG Isotype Control (3900; Cell Signaling Technology) and Mouse IgG Isotype Control (02–6502; Invitrogen, USA) were applied as control respectively. Next, the cells were washed gently with PBS three times and incubated with fluorophore-conjugated secondary antibodies at room temperature for 1 h. The secondary antibodies used were as follows: Alexa Fluor 488 goat anti-rabbit antibody (A-11034; Invitrogen; 1:400) and Alexa Fluor 594 goat anti-mouse antibody (A-11032; Invitrogen; 1:400). DAPI was used to stain cell nuclei. The cells were then rinsed with PBS three times, and antifade mounting medium was added (P0126; Beyotime). All immunostained cells were observed and imaged using a Leica microscope or Leica TCS SP8 confocal system and Leica Application Suite Software. ImageJ 2 software with the Colocalization Finder plugin was used to analyze the colocalization of two proteins in a single cell, and the analysis results for the fluorescence intensity normalized to the maximal intensity of the plot profiles were visualized in Origin 8 software.

### GSC plasma membrane protein extraction

We applied plasma membrane protein extraction kit (ab65400; Abcam) to isolate the cell membrane fraction of GSCs. Based on the manufacturer’s protocol, we purified the cell membrane protein from the total membrane component, as well as the cytosolic fraction for comparison. The plasma membrane fraction western blotting cocktail antibody mixture was applied to detect the fraction markers, such as the sodium-potassium ATPase for the cell membrane, GAPDH for the cytosol and Histone H3 for the nucleus. Anti-GRP78 and anti-BACE2 antibodies were mixed with the antibody cocktail to detect the presence of GRP78 and BACE2 on the GSC surface.

### Detection of csGRP78 by flow cytometry

Cells were dispersed with accutase solution and washed with PBS solution. After blocking nonspecific epitopes with 2% BSA in PBS at room temperature for 30 min, each sample was incubated with 1 μg C38 mouse anti-GRP78 primary antibody for 1 h on ice, with Mouse IgG2b Isotype Control (53,484; Cell Signaling Technology) antibody as the control. Then, the unbound antibodies were rinsed away by three washes with cold PBS, incubating with a PE-conjugated goat anti-mouse IgG secondary antibody (12–4010-82; Invitrogen) at a concentration of 0.25 μg per sample in PBS for 30 min on ice. The samples were washed with PBS, and csGRP78 was detected by C6 flow cytometer and shown as the mean fluorescence intensity in FL2 channel.

### Xenograft model

We constructed GSC267 cells labeled with luciferase (GSC267-luciferase) via lentiviral transfection. All animal experiments were performed with approval from the guidelines of the Institutional Animal Care and Use Committee of Qilu Hospital of Shandong University. 4-week-old male BALB/c nude mice (SLAC laboratory animal Center; Shanghai, China) were bred under specific-pathogen-free conditions at 24 °C on a 12-h day-night cycle, preparing for the establishment of intracranial GSC in situ growth model. Animals with similar condition were randomized as control and experimental groups. After dissociation with accutase solution, 5 × 10^5^ GSC267-luciferase cells were injected intracranially into the right frontal lobe of the mice. The progression of tumorigenesis in vivo was measured by bioluminescence after intraperitoneal injection of 150 mg/kg luciferin, which was detected and imaged with an IVIS Lumina series III ex vivo imaging system (PerkinElmer, USA). We euthanized mice when they showed severe nervous systematic symptoms or became moribund. The survival data were recorded, and the mouse brains were fixed in 4% paraformaldehyde and sectioned to prepare slides for hematoxylin and eosin (H&E) staining.

### Ionizing radiation treatment

For in vitro experiments, GSCs in plates were irradiated with a single dose of 3 Gy and cultured in an incubator for 24 h or 48 h for the subsequent experiments. When irradiation was necessary in animal studies, mice were anesthetized with an animal gas anesthesia system (R640; RWD, China) and treated with 2.5 Gy of radiation continuously for 4 days. Irradiation was performed in the Department of Radiation Oncology at Qilu Hospital of Shandong University.

### Quantitative proteomics (mass spectrometry)

The protein levels in the isotype control and anti-GRP78-blocked groups were quantitatively analyzed via isobaric tags for relative and absolute quantitation (iTRAQ). After 72 h of treatment with 1 μg/ml anti-GRP78 or isotype control antibodies, at least 3 × 10^6^ single GSC267 cells were collected and washed three times with cold PBS solution. Triplicate samples were established for each group; the samples were marked clearly and frozen in liquid nitrogen. Then, total protein extraction, peptide labeling and fractionation, and consistent LC-MS/MS analysis were performed by Novogene (Beijing, China) in an EASY-nLCTM 1200 UHPLC system (Thermo Fisher, USA) coupled to an Orbitrap Q Exactive HF-X mass spectrometer (Thermo Fisher, USA). The protein quantification results were statistically analyzed by the Mann-Whitney test. Significant differences, defined as those with a *P* value of < 0.05 and fold change (FC) of > 1.11 or < 0.9, were used to screen the differentially expressed proteins.

### Bioinformatic analysis

The mRNA sequencing data and corresponding clinical information of total 671 glioma cases were downloaded from The Cancer Genome Atlas (TCGA) database (https://tcga-data.nci.nih.gov/), including 216 WHO II tissues, 239 WHO III tissues, and 156 GBM tissues (Proneural = 18, Classic = 49, Mesenchymal = 67). R version 3.5.1 with the edgeR and pheatmap packages was used to acquire differential gene expression data for GRP78 and BACE2 from the TCGA database. Gene set enrichment analysis (GSEA) was applied to analyze the associations between signaling pathway enrichment and the molecular expression patterns of GRP78 and BACE2 based on the TCGA database. The normalized enrichment score (|NES|) of > 1 and a false discovery rate (FDR) of < 0.25 were considered to indicate significance. The mRNA profiles of MES- and PN-subtype GBM were obtained from samples with subtype classification information in the TCGA database, and the genes with |fold change| of > 2 and adjusted *P* value of < 0.05 were considered the differentially expressed genes between the two subtypes. mRNA profiles of PN (*n* = 6) and MES (*n* = 4) glioma stem-like cell lines were downloaded from the Gene Expression Omnibus (GEO) database (GSE67089), and an adjusted *P* value of < 0.05 was considered to indicate significance.

### Statistical analysis

Statistical analysis was performed using SPSS 20.0 and GraphPad Prism 6. All data are presented as the means ± SDs unless otherwise specified. All cell culture experiments were performed at least in triplicate. Acquired data were certified as normal distribution through Kolmogorove-Smirnov or Shapiro-Wilk test, then Two-tailed *t*-tests and one-way ANOVA were used for comparisons between two independent samples and comparisons among multiple samples, respectively. The Kruskal-Wallis method was applied for comparison between standardized expression data acquired from the TCGA database. The Kaplan-Meier method was applied to analyze the survival data for mice and humans. The Pearson correlation coefficient and R-squared values were calculated using GraphPad Prism to measure the strength of the correlations between genes in the TCGA database. *P* < 0.05 was considered to indicate statistical significance. *P* values are indicated as follows: * *P* < 0.05; ** *P* < 0.01; and *** *P* < 0.001.

## Results

### Total GRP78 expression correlates positively with the MES subtype and contributes to maintenance of the MES phenotype

**Fig. 1:**
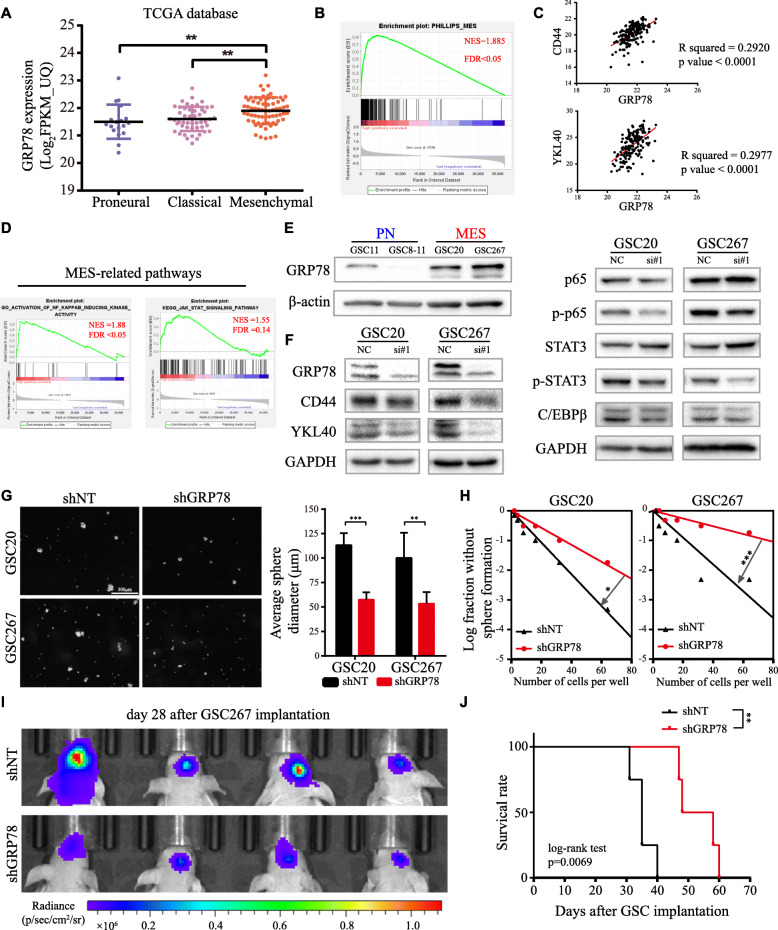
GRP78 in GSCs is associated with MES phenotype. **a** GRP78 mRNA expression level in PN-, CL- and MES-subtype GBMs from TCGA database. **b** GSEA reveals a significantly positive correlation between high expression of GRP78 with MES subtype. **c** Correlation analysis for expression of GRP78 and MES signatures CD44, YKL40 in TCGA database. **d** GSEA analysis reveals high GRP78 is positively enriched in NF-κB and STAT3 pathways. **e** GRP78 protein expression in GSC11, 8–11, 20 and 267. **f** Western blotting for total GRP78 and MES signatures and related pathways in GSC20 and 267 transfected with siGRP78. **g** Images of tumor spheres formation after GSC20 and 267 transfected with lentiviral shGRP78 or shNT and the column plot is the quantified analysis of spheres diameter. Scale bar, 500 μm. **h** In vitro limiting dilution assay for GSC20 and 267 expressing lentiviral shGRP78 or shNT. **i** Bioluminescence of mice implanted with luciferase-labeled GSC267 expressing shGRP78 or shNT. **j** Survival curve of mice in these two groups, tested by log-rank test. Error bar indicates at least three independent experiments and data are shown as mean ± SD. *p < 0.05, **p < 0.01, ***p < 0.001

Through TCGA database, we confirmed that GRP78 mRNA increased with WHO grade and was associated with poor prognosis (Figure S1A, B) and that GRP78 was highest in MES-subtype GBM (Fig. 1a), consistent with GSEA analysis showing that high GRP78 expression was strongly enriched in the MES-subtype gene set (Fig. 1b). In addition, GRP78 expression was positively correlated with the selected MES subtype markers, as well as the enrichment of two important pathways for the MES subtype, STAT3 and NF-κB (p65), but negatively correlated with PN markers (Fig. 1c, d, Figure S1C). Then, we evaluated GRP78 expression in four different GSC lines. As shown in Fig. 1e, GRP78 expression was higher in MES GSCs (GSC20 and GSC267) than in PN GSCs (GSC11 and GSC8–11), and three subclass markers (CD44, SOX2, Olig2) were differentially expressed among these four cell lines (Figure S1D). The MES markers in MES-subtype GSCs were downregulated after interfering with GRP78 expression, consistent with the suppression of the STAT3 and NF-κB pathways and decreased C/EBPβ protein expression (Fig. 1f, Figure S1E). Next, stable knockdown of GRP78 caused significant inhibition of tumorsphere expansion (Fig. 1g) and reduced sphere formation ability in vitro (Fig. 1h). A xenograft model using luciferase-labeled GSC267 cells indicated the suppression of GSC growth in the shGRP78 group versus that in the shNT group (Fig. 1i), which led to an improved survival rate (Fig. 1j). Furthermore, we assessed the effects of radiotherapy on shGRP78 cells. The dual staining apoptosis assay and TUNEL assay results showed that the number of apoptotic tumor cells was the highest in the group treated with the combination of GRP78 knockdown and irradiation (Figure S1F, G), coinciding with the western blotting results for the apoptosis-related protein cleaved PARP (c-PARP) and the radiation injury marker γ-H2AX (Figure S1H). Thus, GRP78 might be an integral molecule for the self-renewal maintenance and radioresistance of MES-subtype GSCs.

### GRP78 relocalizes to the cell surface of GSCs and accounts for the maintenance of the MES phenotype

**Fig. 2:**
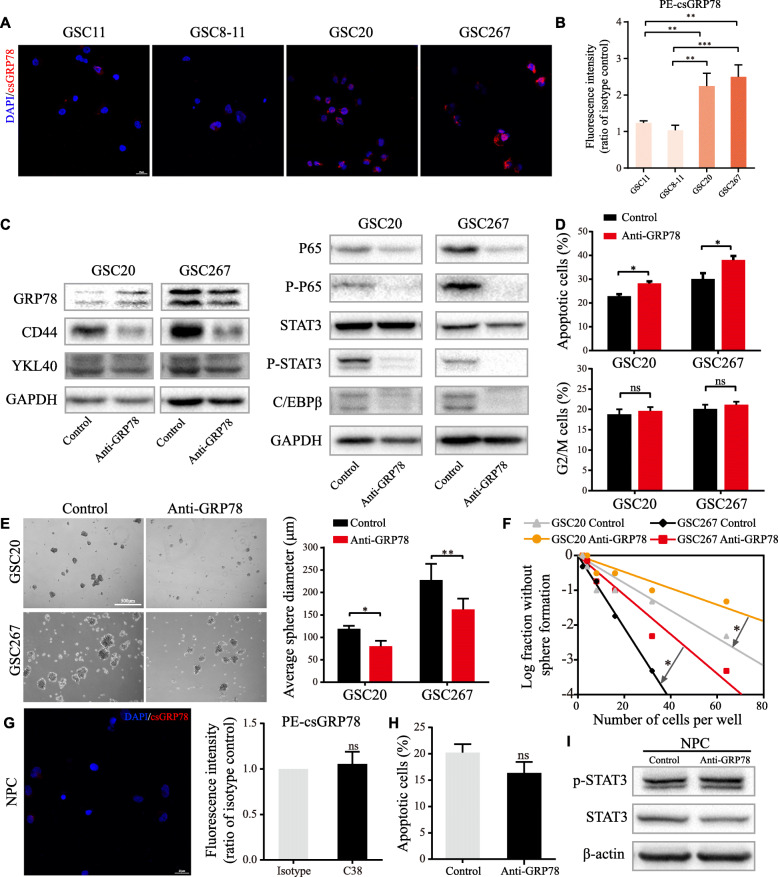
csGRP78 is preferentially expresses in MES GSCs and regulates stemness of GSCs. **a** csGRP78 was detected by nonpermeabilized immunofluorescence in GSC11, 8–11, 20 and 267. Scale bar, 25 μm. **b** csGRP78 in GSCs were quantified by flowcytometry, and the mean fluorescence intensity of PE (FL2 channel) represents the expression of csGRP78. **c** Western blotting for total GRP78 and MES-related signatures and pathways after GSC20 and 267 treated with anti-GRP78. **d** Upper, proportion of apoptotic cells and lower, G2/M phase cells of GSC20 and 267 treated with anti-GRP78. **e** Images of tumor spheres formation after blocking csGRP78 in GSC20 and 267 and quantified with diameter of spheres. Scale bar, 500 μm. **f** In vitro limiting dilution assay for GSC20 and 267 treated with anti-GRP78 **g** Left, nonpermeabilized immunofluorescence of csGRP78 in NPC and right, csGRP78 expression detected by flowcytometry. Scale bar, 25 μm. **h** Apoptosis of NPCs after anti-GRP78 treatment was detected by flowcytometry. **i** Western blotting for phospho-STAT3 and STAT3 protein expression in NPCs after anti-GRP78 treatment. Error bar indicates at least three independent experiments and data are shown as mean ± SD. *p < 0.05, **p < 0.01, ***p < 0.001

First, immunofluorescence staining without permeabilization and flow cytometry clearly showed that csGRP78 (csGRP78) was expressed in GSC lines and more abundant in the MES-subtype lines GSC20 and GSC267 (Fig. 2a, b). We also demonstrated the existence of GRP78 on the surface of GSCs through the isolation and purification of plasma membrane proteins (Figure S2A). The expression of csGRP78 decreased partly with siGRP78 transfection (Figure S2B). To clarify the function of csGRP78 in MES GSCs, we applied an anti-GRP78 antibody, which recognizes an epitope located near the C-terminus sequence, to block csGRP78. After 72 h of treatment with the anti-GRP78 antibody, MES-specific markers and crucial pathways were markedly downregulated without obvious changes in the total GRP78 protein level (Fig. 2c). In addition, blocking csGRP78 in MES GSCs induced apoptosis, consistent with the results of previous studies [13] (Fig. 2d; Figure S2C, D). As expected, blocking csGRP78 noticeably influenced GSC tumorsphere formation ability and probability (Fig. 2e, f). Taken together, these results indicate that the relocalization of GRP78 to the cell surface occurs in GSCs, especially in the MES subclass, which might play a predominant role in the maintenance of the MES subtype.

### Cell surface blockade specifically targets MES-subtype GSCs but not neural progenitor cells (NPCs)

As a normal type of stem cell in the human nervous system, NPCs share some similarities with GSCs [21]. Thus, the specificity that restrains the stemness of tumor stem cells without apparent effects on normal stem cells is an indispensable consideration for a therapeutic strategy. We used immunofluorescence staining and flow cytometry to detect csGRP78 on the NPC plasma membrane and found no positive results (Fig. 2g). Treatment with the same antibody used for GSCs did not significantly inhibit neural sphere formation or apoptosis in NPCs (Figure S2E, Fig. 2h), and western blotting demonstrated that antibody treatment did not suppress the activation of STAT3 in NPCs (Fig. 2i). These data suggest that blocking csGRP78 with an antibody effectively disrupts MES GSCs but has no detectable effects on the stemness and viability of NPCs.

### Cell surface blockade sensitizes MES GSCs to radiotherapy in vitro and in vivo

**Fig. 3:**
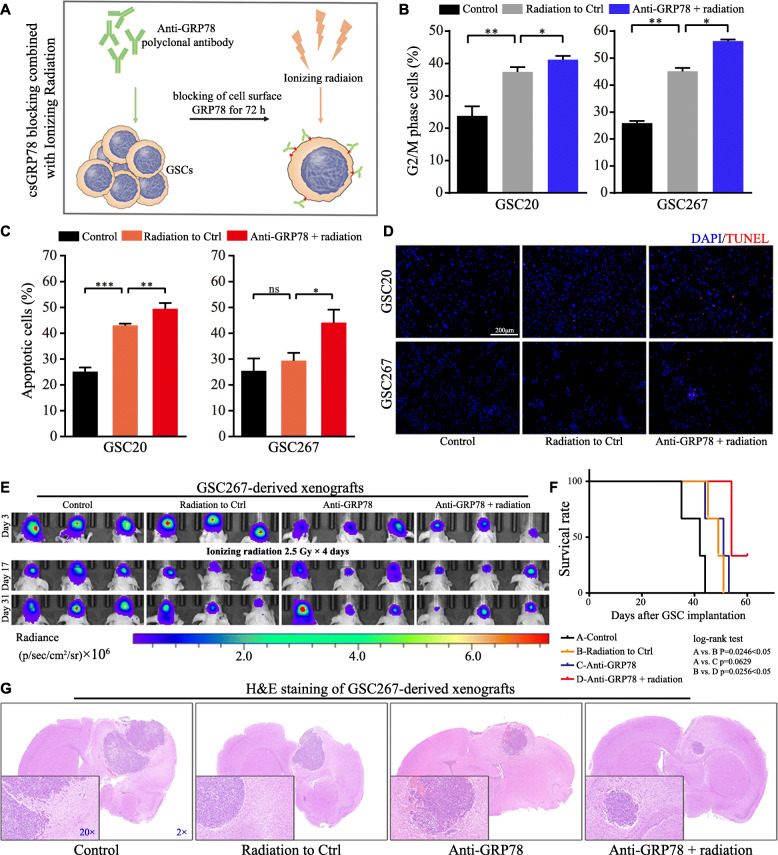
Blockade of csGRP78 impairs radioresistance in MES-subtype GSCs. **a** Work flow depicts the combination of anti-GRP78 and radiation for the consequent experiments. **b** Cell cycle analysis for G2/M phase cells of GSC20 and 267 after 3 Gy radiation only or combined with anti-GRP78 pretreatment. **c** and **d** Apoptosis of GSC20 and 267 after 3 Gy radiation only or combined with anti-GRP78 pretreatment was detected by flowcytometry and TUNEL staining, as the red dot presenting the radiation damage. Scale bar, 200 μm. **e** Bioluminescence imaging for mice implanted with luciferase-labeled GSC267 with treatments of isotype control, anti-GRP78, and/or radiation at the indicated time points. The radiation for animals was started at day 3 after GSCs implantation. **f** Survival data of mice in every group, and the statistical significance was tested by log-rank test. **g** Represented images of H&E staining for a subgroup of animal sacrificed simultaneously in each group. The magnification was marked in blue number. Error bar indicates at least three independent experiments and data are shown as mean ± SD. *p < 0.05, **p < 0.01, ***p < 0.001

Radiotherapy resistance is considered a characteristic of MES-subtype GSCs in contrast to the PN subtype. Thus, we determined whether blocking csGRP78 could enhance the therapeutic efficacy of radiotherapy (Fig. 3a). Although monotherapy with 3 Gy radiation delivered to GSC20 and GSC267 cells caused G2/M cell cycle arrest and apoptosis, the combination of radiation with antibody treatment escalated the harmful outcome of radiation (Fig. 3b, c; Figure S3A, B). The results of TUNEL and western blotting for c-PARP and γ-H2AX also indicated increased injury after radiotherapy (Fig. 3d, Figure S3C). Then, we established orthotopic xenografts to prove that the combination of antibody treatment and radiotherapy prolongs animal survival. For anti-GRP78 treatment, we pretreated GSC267 cells with 1 μg/ml anti-GRP78 antibody for 72 h before implantation, and radiation was administered 3 days after implantation. The bioluminescence images and survival analysis results showed that monotherapy with anti-GRP78 reduced GSC-derived tumor growth initially but failed to prolong survival over the 60-day observation period. Radiotherapy only partially restrained tumor progression, but in regard to the combined therapy, the animals received enhanced survival benefits (Fig. 3e, f). H&E staining of a subset in each group provided a histological demonstration of the tumor volume (Fig. 3g).

### Blocking csGRP78 changes the protein expression profile in GSC267 cells, including that of proteins encoded by MES phenotype-associated genes

**Fig. 4:**
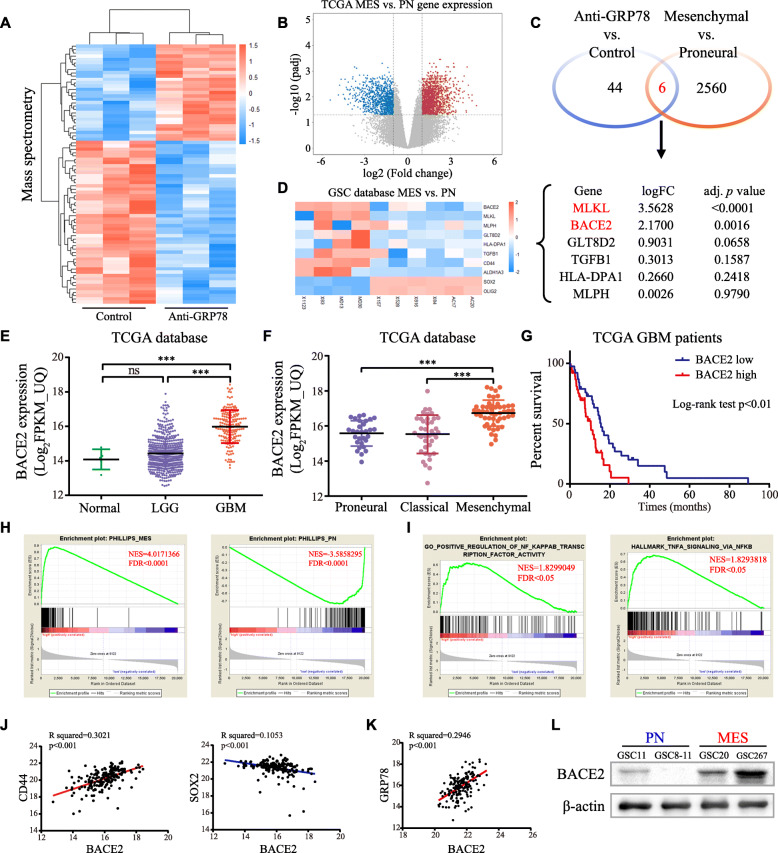
Screen BACE2 as a candidate of csGRP78 downstream molecule. **a** Heat map shows the differential proteins acquired by mass spectrometry. Anti-GRP78 versus Isotype, blue indicates downregulation while red indicates upregulation. **b** mRNA profile distinguishes PN GBMs from MES GBMs in TCGA database. **c** Compared downregulated proteins in anti-GRP78 versus isotype with upregulated genes in MES-subtype GBMs, overlapping total 6 genes. **d** Statistical analysis for differential expression of these 6 candidates in mRNA profiling of MES and PN GSCs (GSE67089). **e** BACE2 mRNA expression in normal tissues, low-grade glioma (LGG) and GBM from TGCA database. **f** BACE2 mRNA expression in TCGA database grouped by subtype. **g** Survival curve and log-rank test for GBM patients stratified by the BACE2 expression from TCGA database. **h** GSEA indicates expression of BACE2 positively correlated with MES subtype, while negatively with PN subtype. **i** GSEA shows BACE2 has the strong correlation with NF-κB pathway. **j** Correlation between expression of BACE2 with selected MES and PN signatures in TCGA database. **k** Correlation analysis for expression of BACE2 and GRP78 in TCGA database. **l** western blot analysis for BACE2 in GSC11, 8–11,20 and 267. *p < 0.05, **p < 0.01, ***p < 0.001

To further investigate the mechanism by which csGRP78 participates in MES phenotype maintenance, we performed quantitative proteomics to search for downstream proteins in GSC267 cells, which express higher levels of total and cell surface GRP78. After csGRP78 was blocked, the expression of a total of 85 proteins changed (32 were upregulated, while 53 were downregulated). Interestingly, membrane proteins accounted for the largest proportion of differentially expressed proteins (Fig. 4a; Figure S4A, B). The volcano plot indicates the differentially expressed genes between MES-subtype and PN-subtype GBM in the TCGA database (Fig. 4b). Comparison of the proteins reduced by blocking csGRP78 to genes upregulated in MES-subtype tumor tissues revealed 6 overlapping molecules with the potential to regulate the MES phenotype of GSCs. Furthermore, we used the mRNA profile data to analyze the fold change (FC) and significance value between two subtypes of GSC lines, identifying the potential molecules in the context of GSCs. Through these steps, BACE2 and mixed lineage kinase domain-like pseudokinase (MLKL) were screened out as candidates (Fig. 4d). However, the survival analysis stratified by MLKL expression showed no correlation between MLKL mRNA expression and prognosis in GBM patients (Figure S4C). Therefore, BACE2 is probably a downstream molecule regulated by csGRP78.

### BACE2 is strongly correlated with the MES subtype and poor prognosis

Based on the TCGA database, GBM samples expressed higher BACE2 expression than normal tissues or low-grade gliomas (Fig. 4e), and BACE2 mRNA levels were significantly higher in the MES subtype (Fig. 4f). In GBM patients, higher BACE2 expression was associated with shorter survival times (Fig. 4g). Then, we analyzed the correlation between BACE2 expression and published MES or PN signature gene sets [6]. As expected, the MES and PN gene sets were positively and negatively enriched, respectively, with high BACE2 expression (Fig. 4h). BACE2 expression was also positively correlated with NF-κB pathway activity (Fig. 4i). Similarly, the correlation between BACE2 and selected subtype markers was positive for MES markers and negative for PN markers (Fig. 4j, Figure S4D). In addition, BACE2 correlated positively with GRP78 expression, and BACE2 protein was highly expressed in MES GSCs, especially in GSC267 cells, which have the highest abundance of both total and cell surface GRP78 (Fig. 4k, l). Taken together, BACE2 is closely related to GRP78 and might play an important role in the biological behaviors of MES GSCs.

### BACE2 is involved in maintaining the mesenchymal phenotype of GSCs

**Fig. 5:**
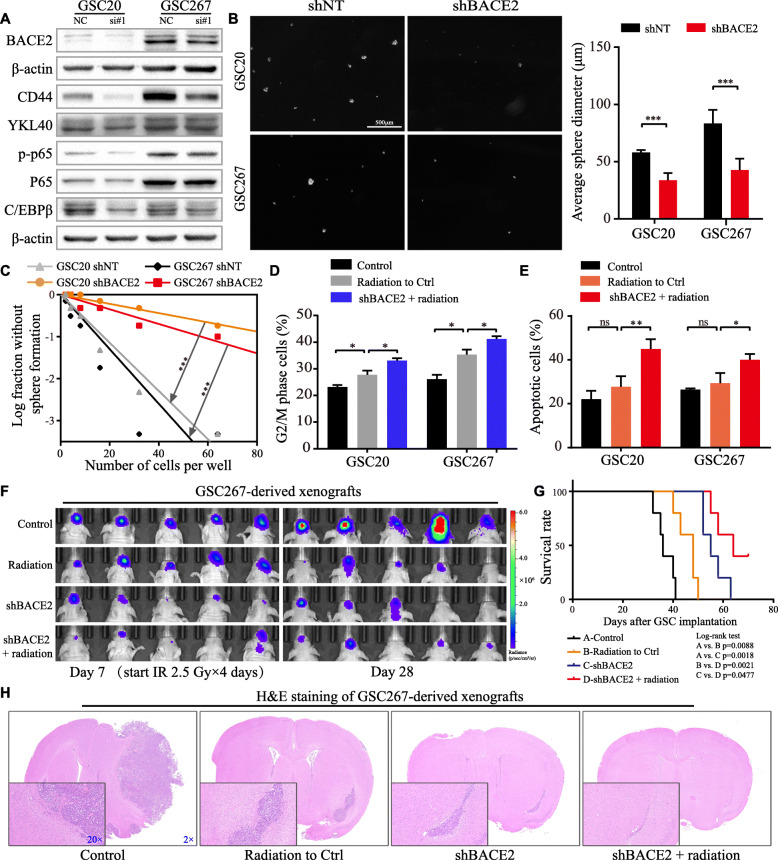
Knockdown of BACE2 in MES GSCs restrains stemness and radioresistance. **a** Western blotting analysis for MES-related signatures CD44, YKL40 and pathways p65, p-p65 and C/EBPβ after silencing BACE2 in GSC20 and 267. **b** Left, images of spheres formation assay after GSC20 and 267 transfected with lentiviral shBACE2 or shNT and right, results were quantified with diameter of tumor spheres. Scale bar, 500 μm. **c** In vitro limiting dilution assay for GSC20 and 267 expressing lentiviral shBACE2 or shNT. **d** Cell cycle analysis for G2/M phase cells of GSC20 and 267 in control, 3 Gy radiation only or combined with shBACE2 groups. **e** Flowcytometry detected apoptosis of GSC20 and 267 in control, 3 Gy radiation only or combined with shBACE2 groups. **f** Bioluminescence imaging for xenografts grouped as control, shBACE2 and/or radiation at the indicated time points. The radiation was delivered at day 7 after GSCs implantation. **g** Survival curve for each group, and the statistical significance was tested by log-rank test. **h** Represented images of H&E staining in each group. The magnification was marked in blue number. Error bar indicates at least three independent experiments and data are shown as mean ± SD. *p < 0.05, **p < 0.01, ***P < 0.001

To experimentally investigate the function of BACE2 in MES GSCs, we utilized two independent siRNA sequences to knock down BACE2. Western blotting analysis indicated downregulation of MES-related signatures and the signaling pathway components p-p65 and C/EBPβ (Fig. 5a, Figure S5A). Moreover, knockdown of BACE2 dramatically decreased the self-renewal of MES GSCs (Fig. 5b, c). The results of radiotherapy experiments demonstrated that the combination of radiotherapy with shBACE2 resulted in a greater proportion of cells with G2/M phase arrest and apoptotic cells, suggesting that shBACE2 sensitized MES GSCs to radiotherapy (Fig. 5d, e; Figure S5B, C). TUNEL and western blotting for c-PARP and γ-H2AX supported the same conclusion (Figure S5D, E). GSC267-derived orthotopic xenografts were applied to determine the effects of targeting BACE2 in MES-subtype GSCs on tumorigenesis and radioresistance in vivo. Bioluminescence imaging 7 days after GSC implantation as well as the survival data demonstrated that BACE2 knockdown inhibited tumor growth compared with that in the shNT group, and radiotherapy was administered at this time. Subsequent imaging and survival analysis revealed that the combination of radiotherapy with BACE2 knockdown prolonged animal survival, which was better than each monotherapy group (Fig. 5f, g). H&E staining from a subset of mice presented the tumor volumes at the same time point (Fig. 5h). The knockdown efficiency of lentiviral shGRP78 and shBACE2 was detected by western blotting (Figure S5F). Collectively, these results demonstrate that targeting BACE2 strongly inhibits stemness and increases the efficacy of radiotherapy in MES-subtype GSCs; hence, BACE2 could partially account for the function of csGRP78 in MES-subtype GSCs.

### csGRP78 is coexpressed with BACE2 on MES-subtype GSCs and regulates their lysosomal degradation

**Fig. 6:**
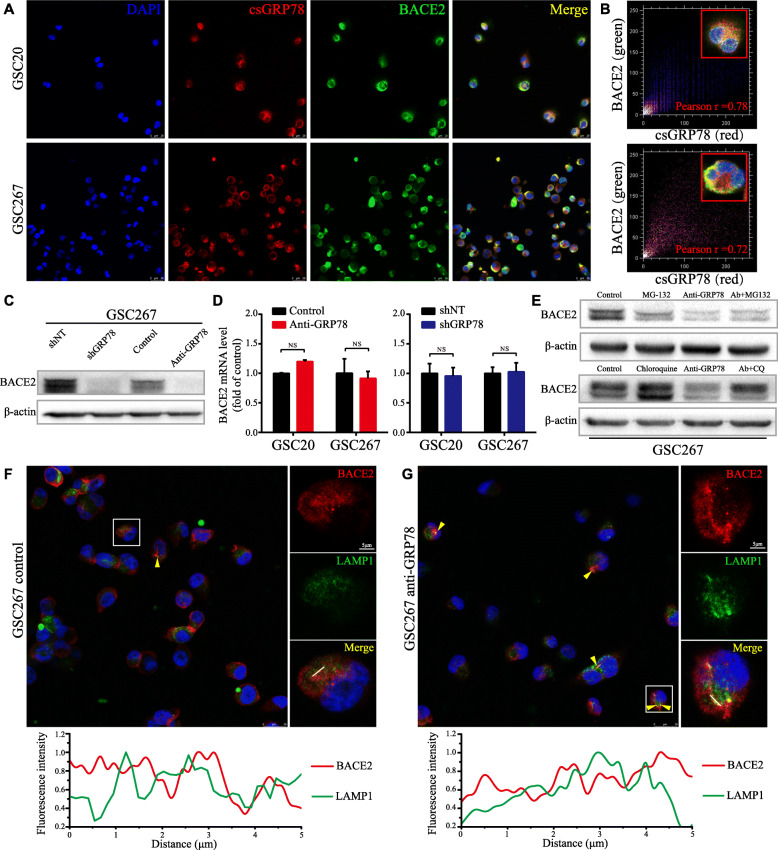
Blocking csGRP78 induces lysosomal degradation of BACE2. **a** Co-immunofluorescence staining of csGRP78 (red) and BACE2 (green) in GSC20 and 267. Scale bar, 25 μm. **b** Colocalization analysis for co-expression of BACE2 and csGRP78 in cell membrane of GSC20 and 267 using colocalization finder plugin. **c** Western blotting for BACE2 in GSC267 with anti-GRP78 treatment for 72 h or lentiviral shGRP78 expressing. **d** BACE2 RNA level in GSC20 and 267 was detected by qRT-PCR with anti-GRP78 treatment or lentiviral shGRP78 expressing, GADPH as the reference gene. **e** Western blotting for BACE2 protein in GSC267 that treated with MG-132, Chloroquine (CQ) or anti-GRP78, respectively, and co-treatment of anti-GRP78 with MG-132 or CQ. **f** and **g** Upper, the confocal microscopy images for overview and splitting channel of BACE2 (red) and LAMP1 (green). The yellow triangles indicate the overlapped regions (yellow). Lower, the analysis plot for the fluorescence intensity of two channels. Red line for BACE2 and green line for LAMP1. Scale bar, 5 μm, and 25 μm for the large field of view. Error bar indicates at least three independent experiments and data are shown as mean ± SD

Through nonpermeabilized immunofluorescence staining, we found that csGRP78 was coexpressed with BACE2 on the cell surface (Fig. 6a). Pearson’s correlation coefficient was measured, showing a strong correlation between csGRP78 and BACE2 in MES-subtype GSCs (Fig. 6b), and BACE2 protein expression was decreased after blockade of csGRP78 with the anti-GRP78 antibody in GSC20 and GSC267 cells and even stable silencing of GRP78 in GSC267 cells (Fig. 6c, Figure S6A). We next investigated the mechanism by which csGRP78 regulates BACE2 expression. First, we measured BACE2 mRNA expression via qRT-PCR after csGRP78 blockade or lentiviral GRP78 knockdown. Neither antibody-mediated blockade nor constant GRP78 silencing resulted in a significant change in BACE2 mRNA expression, indicating that the posttranscriptional process was dominantly involved (Fig. 6d). Previous research demonstrated that BACE2 degradation was lysosome-dependent and that the half-life of BACE2 was approximately 20 h [22]. Thus, we sought to determine whether BACE2 was regulated in a lysosomal manner in MES-subtype GSCs. We treated GSC267 cells with the proteasomal inhibitor MG-132 (10 μM) for 10 h or the lysosomal inhibitor chloroquine (CQ, 20 μM) for 10 h using the same dose of solvent as a control. As a result, chloroquine increased the expression of BACE2, while MG-132 caused no noticeable change. Moreover, blocking csGRP78 for 48 h markedly downregulated BACE2 protein expression, which could be rescued by chloroquine but not MG-132 (Figure S6B, C; Fig. 6e). For further clarification, we observed the colocalization of BACE2 and LAMP1, a protein marker for lysosomes, using coimmunofluorescence staining and colocalization analysis under anti-GRP78 treatment. Consistent with the results presented above, blocking csGRP78 increased the distribution of BACE2 in lysosomes, manifested as a larger overlapping region and greater coherence of the two channels in the image analysis plots. (Fig. 6f, g). Collectively, these results indicate that blocking csGRP78 in MES-subtype GSCs can disrupt the stabilization of BACE2, leading to its lysosomal degradation.

### Radioresistance acquired from GRP78 overexpression can be blocked by anti-GRP78 antibodies

**Fig. 7:**
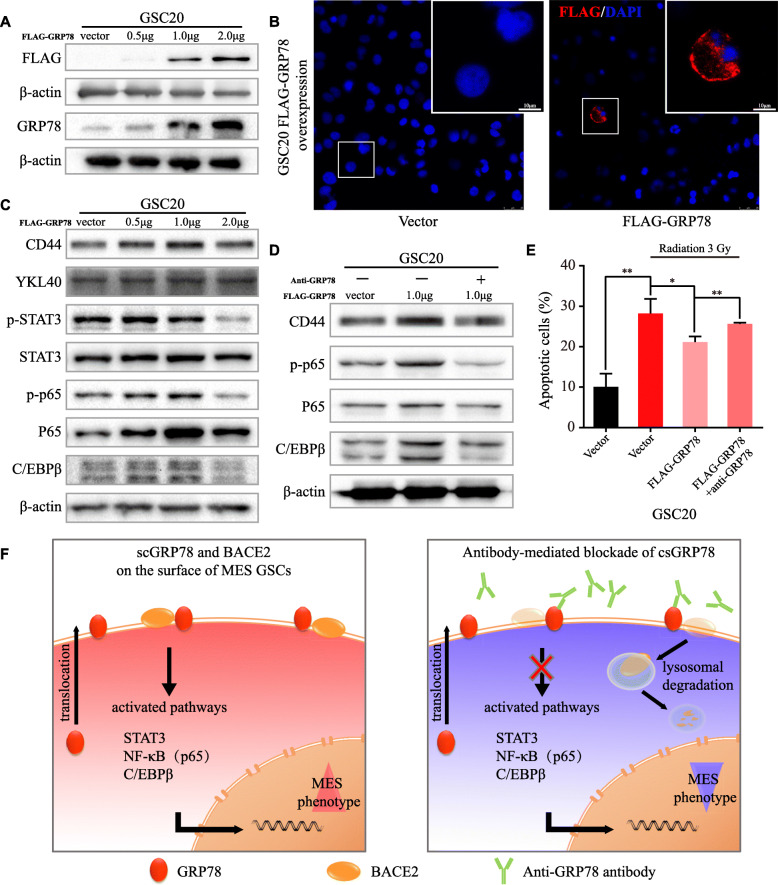
Anti-GRP78 blockades exogenous GRP78-derived reinforcement of MES phenotype. **a** Western blotting for FLAG tag and total GRP78 in GSC20 transduced with vector control or WT FLAG-GRP78. **b** Nonpermeabilized Immunofluorescence staining of FLAG (red) in GSC20 transfected with FLAG-GRP78, taking vector as control. Scale bar, 10 μm, and 25 μm for the large field of view. **c** Western blotting for MES-related signatures CD44, YKL40 and pathways STAT3, p-STAT3, p65, p-p65 and C/EBPβ in GSC20 transfected with FLAG-GRP78 or vector control. **d** Western blotting for CD44, p65, p-p65 and C/EBPβ in GSC20 expressing FLAG-GRP78 with or without anti-GRP78 cotreatment for 48 h. **e** Flowcytometry demonstrated the apoptosis of GSC20 with 3 Gy radiation, which transfected with vector or FLAG-GRP78, with or without anti-GRP78 cotreatment. **f** Summary diagram for the possible mechanisms of csGRP78 playing an essential role in maintaining MES phenotype and targeting csGRP78 impairs BACE2 stabilization via lysosome-dependent degradation in MES subtype GSCs. Error bar indicates at least three independent experiments and data are shown as mean ± SD. *p < 0.05, **p < 0.01, ***p < 0.001

To verify the ability of csGRP78 in MES-subtype GSCs to obtain stemness and radioresistance, we examined MES-related signatures and signaling pathways after transfection of GSC20 cells with the full-length WT FLAG-GRP78 plasmid. The total GRP78 protein level was elevated in accordance with that of the exogeneous FLAG tag (Fig. 7a), and nonpermeabilized immunofluorescence for the FLAG tag indicated that the overexpressed GRP78 could relocalize to the cell membrane (Fig. 7b). For the optimal dose to reinforce the MES phenotype, transfection with 1 μg of FLAG-GRP78 showed the most significant increase in CD44, p-p65 and C/EBPβ, as well as BACE2 protein expression (Fig. 7c, Figure S7A). Next, by transfecting GSC20 cells with 1 μg of FLAG-GRP78, we assessed whether protein expression changes and radioresistance could be reversed through anti-GRP78 treatment. Western blotting showed that the elevation of CD44, p-p65 and C/EBPβ levels was decreased by anti-GRP78 cotreatment (Fig. 7d). The apoptosis assay after radiation demonstrated that overexpression of GRP78 decreased apoptosis compared with radiation alone and that the acquired resistance was alleviated by blocking csGRP78 (Fig. 7e). We also overexpressed GRP78 in PN-subtype GSC8–11 cells, which increased p-p65, p65 and C/EBPβ (Figure S7B, C) but failed to increase the expression of the MES signature markers CD44 and YKL40 (data not shown). Collectively, these results indicate that increasing csGRP78, which can be effectively targeted by antibodies, in MES-subtype GSCs reinforces mesenchymal characteristics. Finally, a summary diagram of the mechanism by which csGRP78 maintains the self-renewal and radioresistance of MES GSCs is presented (Fig. 7f).

## Discussion

Prior studies have uncovered the high intertumoral and intratumoral heterogeneity of GBMs, which emphasizes the impossibility associated with unified treatment for GBM. Based on the diversity of the transcriptional, genotypic, and epigenetic states, GBM as well as GBM-derived GSCs can be classified into the PN, CL and MES subtypes [2, 3, 6]. It is well known that PN GSC transition to MES GSCs often occurs under certain circumstances, especially upon recurrence, which is defined as high radioresistance and poor prognosis [23, 24]. Moreover, recent single-cell expression profiling studies further demonstrate that multiple GSC cellular states may interconvert with each other, including MES GSCs [25]. Therefore, targeting MES GSCs is an inevitable consideration for an effective therapeutic strategy for GBM. Here, we explored whether csGRP78, compared to PN GSCs and NPCs, was highly expressed in MES GSCs. Further study indicated that targeting csGRP78 might be a promising adjuvant strategy to prohibit the malignant progression and recurrence of GBM.

The function of cytosolic GRP78, which influences cancer biological behaviors, has been thoroughly studied [9, 26]. However, studies of GSCs are limited. Thanh-Tuan Huynh et al. reported that the GRP78/miR-205 axis was associated with the self-renewal and radioresistance of CD133-positive GSCs [27]. In our study, we verified a positive correlation between GRP78 and the MES phenotype by using TCGA data and silencing GRP78 in MES-subtype GSCs (Fig. 1). As suggested by the findings of Yuan-Li Tsai et al., ER stress can relocalize the ER chaperone GRP78 from the cytosol to the cell surface via SRC activation [28]; thus, csGRP78 expression corresponding to the total GRP78 level might be a reasonable mechanistic explanation for MES phenotype maintenance.

Accumulating studies have shown that csGRP78 participates in the control of tumor biological behaviors and signaling [29] and that blocking csGRP78 on C-terminus or N-terminus epitopes leads to cancer cell death and increased radiosensitivity in pancreatic cancer [13, 30, 31]. In this work, antibodies targeting the C-terminus of GRP78 inhibited the self-renewal ability and radioresistance of MES GSCs and suppressed STAT3, NF-κB (p65) and C/EBPβ (Figs. 2, 3). These pathways have been fully proven to be closely related to the transformation and maintenance of the MES phenotype [7, 32, 33]. Surprisingly, antibody pretreatment of GSC-derived xenografts failed to prolong animal survival over the long-term observation period. We hypothesized that a single-dose pretreatment with the antibody was probably not lethal to GSCs and that the blocking effects failed without continuous dosing. As well described by previous articles, factors triggering ER stress, such as hypoxia and ionizing radiation, are more common in the microenvironment of tumors than in that of normal brain cells [34, 35], and these factors are considered to cause MES transition and maintenance [8, 36]. Based on these data, csGRP78 is more likely to relocalize in MES-subtype GSCs and plays a specific role in MES phenotypic maintenance, which is reported in our findings. Therefore, targeting csGRP78 in patients with glioblastoma could be a novel specific therapeutic strategy aimed at MES GSC-derived resistance to therapy.

Antibody-mediated posttranscriptional regulation is a common mechanism underlying changes in downstream molecules. It is effective to use specific antibodies to induce endocytosis of oncogenic proteins or disturb the balance of targeted antigen recycling between endosomes and lysosomes [37, 38]. Our results reveal the potential of csGRP78 in regulating membranous protein expression in GSCs. One of the downstream proteins is BACE2, which was found to be degraded in a lysosome-dependent manner mediated by csGRP78 (Figs. 4, 6).

BACE2 is a molecule that has been well studied in neurodegenerative diseases as the enzyme cleaving APP, the precursor of amyloid-β peptides (Aβ), a hallmark of Alzheimer’s disease [39]. We previously noted that BACE2 is highly expressed in GBMs and positively modulates NF-κB signaling to promote invasiveness and proliferation [40]. In this work with GSCs, we demonstrated the indispensable function of BACE2 in MES phenotype maintenance through NF-κB and C/EBPβ. However, the mechanism by which BACE2 modulates NF-κB signaling in MES GSCs is unclear and requires further investigation.

## Conclusion

Conclusively, csGRP78 was preferentially expressed in MES-subtype GSCs, demonstrating the capacity of regulating MES-related proteins in a lysosome-dependent manner. Targeting csGRP78 in MES GSCs successfully downregulated MES phenotypic signatures and pathways, leading to the suppression of self-renewal and radioresistance of tumor cells without hampering NPC stemness. Therefore, targeting csGRP78 in MES GSCs might be a promising approach to overcome the recurrence and therapeutic resistance of GBMs.

## Supplementary Information

**Additional file 1: Table S1.** The detailed information of primers’ sequence for qPCR and sequences of RNA interfering applied.

**Additional file 2.** Supplementary figures and legend.

## Data Availability

The datasets used and/or analysed during the current study are available from the corresponding author on reasonable request.

## Abbreviations

GRP78: Glucose-Related Protein 78
GSC: Glioma-Stem Cell
MES: Mesenchymal
PN: Proneural
CL: Classical
GBM: Glioblastoma Multiforme
BACE2: β-site APP-cleaving enzyme 2
TCGA: The Cancer Genome Atlas
NPC: Neural Progenitor Cell
GEO: The Gene Expression Omnibus
GSEA: Gene Set Enrichment Analysis
IHC: Immunohistochemistry
STAT3: Signal Transducer and Activator Of Transcription 3
NF-κB: Nuclear factor kappa B
C/EBPβ: CCAAT Enhancer Binding Protein Beta
TUNEL: TdT-mediated dUTP Nick-End Labeling
WT: Wild type
PARP: Poly (ADP-Ribose) Polymerase
