# Supplementary material for: Cell surface GRP78 regulates BACE2 via lysosome-dependent manner to maintain mesenchymal phenotype of glioma stem cells
Source: J Exp Clin Cancer Res. 2021 Jan 7;40:20. doi: 10.1186/s13046-020-01807-4 (PMC7791784; doi:10.1186/s13046-020-01807-4)
Supplement: Supplementary file 1 — Additional file 1: Table S1. The detailed information of primers’ sequence for qPCR and sequences of RNA interfering applied. [file 13046_2020_1807_MOESM1_ESM.pdf]

Additional file 1: Table S1

Table S1: Information of the qPCR primer sequences and RNA interfering sequences

**qPCR primers**	**Sequence**	**company**
Homo-BACE2 (F)	5′CGTTTTCTCCATGCAGATGAGTGT3′	BioSune Biotechnology, Shanghai, China
Homo-BACE2 (R)	5′CCTCCGTTGGTCCCCAGATC3′	
Homo-GAPDH (F)	5’GCACCGTCAAGGCTGAGAAC3’	
Homo-GAPDH (R)	5’TGGTGAAGACGCCAGTGGA3’	
**RNA interfering**	**Target sequence**	**company**
siGRP78#1	5’GGAGCGCAUUGAUACUAGA3’	GenePharma, Shanghai, China
siGRP78#2	5’GGGCAAAGATGTCAGGAAA3’	RiboBio, Guangzhou, China
siBACE2#1	5’GCCUUUCUCAACAGAGGAU3’	GenePharma, Shanghai, China
siBACE2#2	5′ACAGAGAGGUCUAGCACAU3’	GenePharma, Shanghai, China
Lentiviral shGRP78	5’GGAGCGCAUUGAUACUAGA3’	GeneChem, Shanghai, China
Lentiviral shBACE2	5′ACAGAGAGGUCUAGCACAU3’	GenePharma, Shanghai, China

Negative control was provided by each company respectively
