# Supplementary material for: Cell surface GRP78 regulates BACE2 via lysosome-dependent manner to maintain mesenchymal phenotype of glioma stem cells
Source: J Exp Clin Cancer Res. 2021 Jan 7;40:20. doi: 10.1186/s13046-020-01807-4 (PMC7791784; doi:10.1186/s13046-020-01807-4)
Supplement: Supplementary file 2 — Additional file 2. Supplementary figures and legend. [file 13046_2020_1807_MOESM2_ESM.pdf]

Supplementary figures

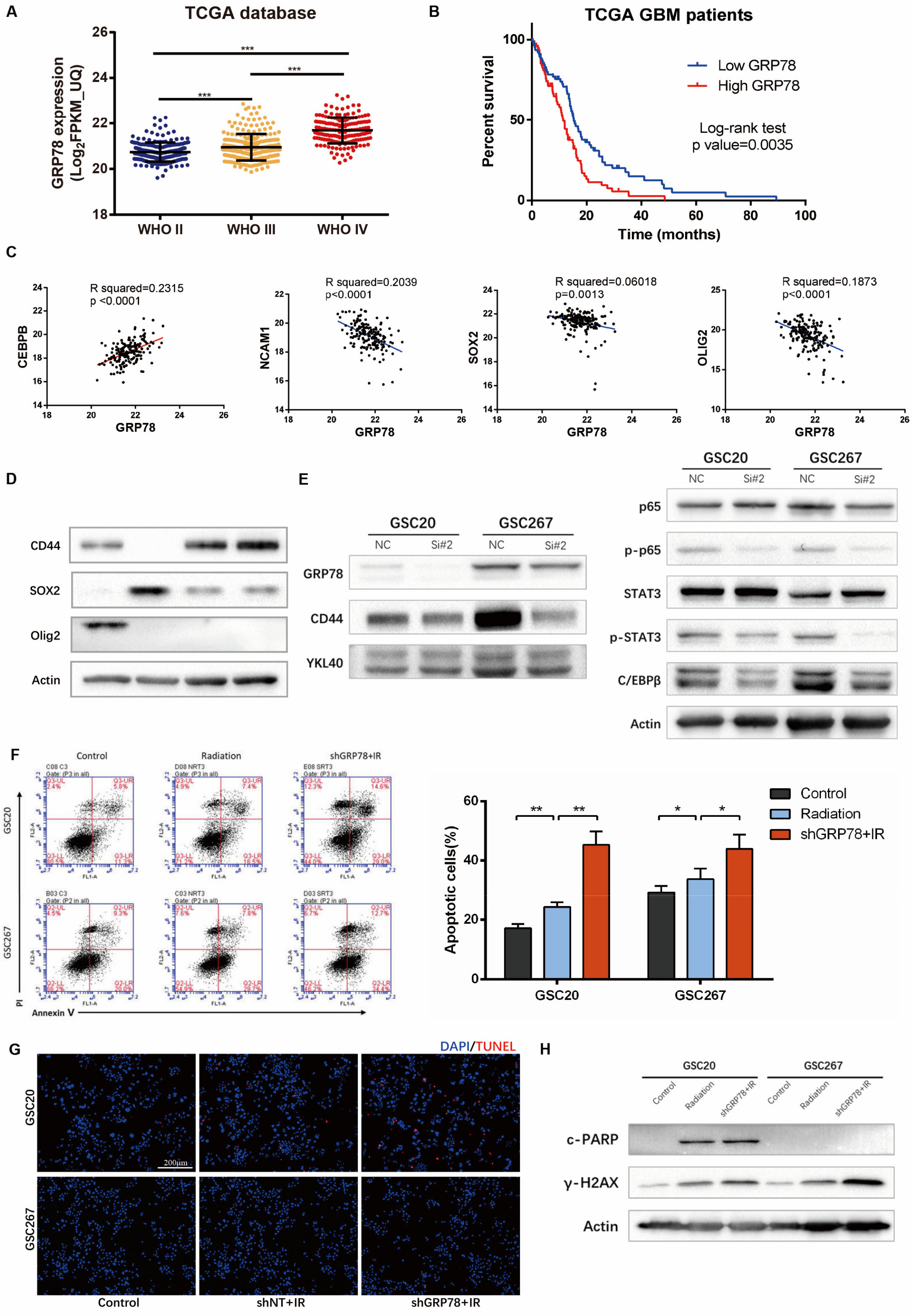

Supplementary figure 1

A. GRP78 mRNA expression in glioma grouped by WHO grade from TCGA database.

B. Survival curve and log-rank test for TCGA GBM patients with low or high expressed GRP78.

C. Same as Figure 1C, correlation of GRP78 expression with CEBPB, NCAM1, SOX2 and OLIG2 in TCGA database for glioma.

D. Protein expression of CD44, SOX2 and Olig2 in four GSC cell lines.

E. Same blot as Figure 1F using another siGRP78 sequence targeted total GRP78.

F. AnnexinV-PI dual staining detected apoptosis in GSC20 and 267 with treatment of control, radiation combined with or without shGRP78. (IR = ionizing radiation)

G. TUNEL staining for GSCs grouped by same treatments mentioned above. Scale bar, 200 μm.

H. Western blotting for cleaved-PARP and γ-H2AX.

Error bar indicates at least three independent experiments. *p <0.05, **p <0.01, ***p <0.001.

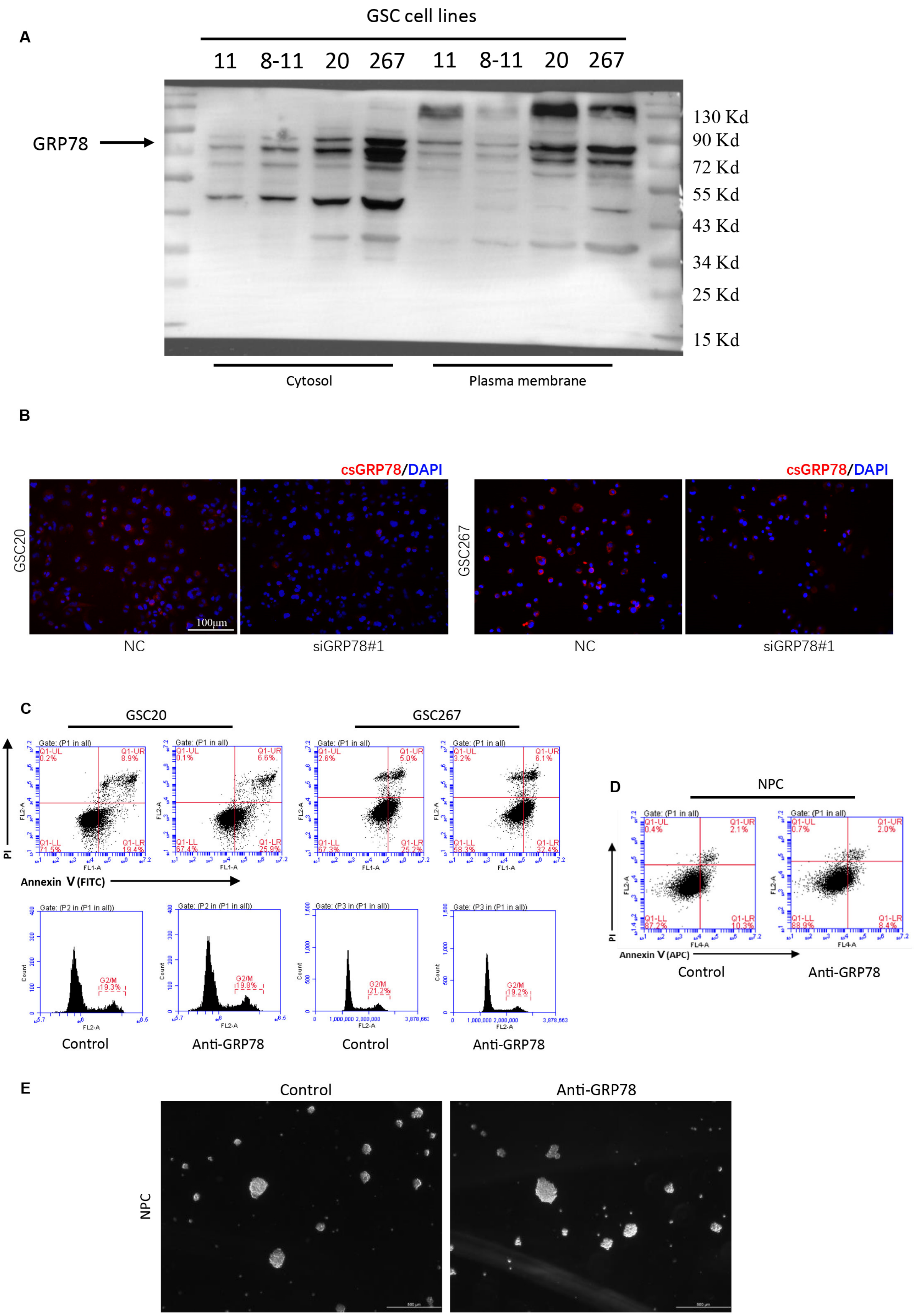
A
GSC cell lines
11
8-11
20
267
11
8-11
20
267
GRP78
130 Kd
90 Kd
72 Kd
55 Kd
43 Kd
34 Kd
25 Kd
15 Kd
Cytosol
Plasma membrane
B
csGRP78/DAPI
GSC20
100μm
NC
siGRP78#1
csGRP78/DAPI
GSC267
NC
siGRP78#1
C
GSC20
GSC267
PI
Annexin V (FITC)
Control
Anti-GRP78
Control
Anti-GRP78
D
NPC
PI
Annexin V (APC)
Control
Anti-GRP78
E
Control
Anti-GRP78
NPC

Supplementary figure 2

A. Isolation and purification of plasma membrane fraction showed GRP78 is highly expressed in plasma membrane of MES GSCs, using sodium-potassium ATPase as the marker of plasma membrane.

B. The nonpermeabilized immunofluorescence for scGRP78 in MES GSCs with siGRP78#1 targeting total GRP78. Scale bar, 100 μm.

C. C6 flow cytometer plots of Figure 2D.

D. C6 flow cytometer plots of Figure 2H.

E. Images of neurosphere formation assay for NPCs with or without anti-GRP78 treatment. Scale bar, 500 μm.

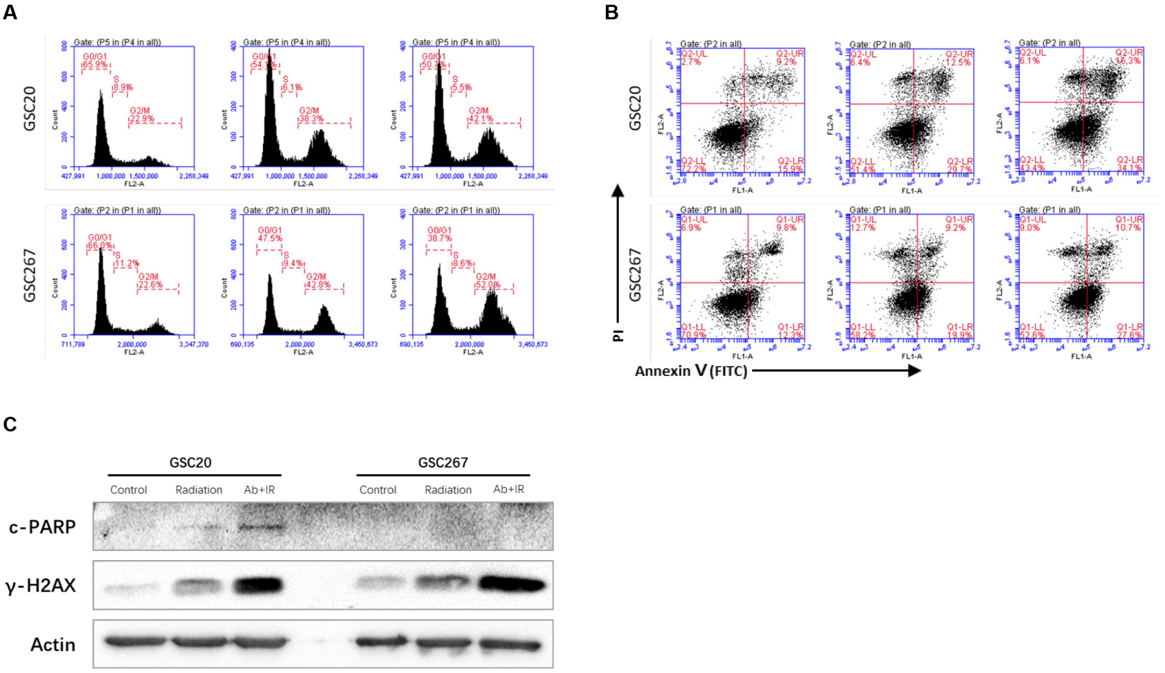

Supplementary figure 3

A. C6 flow cytometer plots of Figure 3B.

B. C6 flow cytometer plots of Figure 3C.

C. Western blotting for cleaved-PARP and γ-H2AX in MES GSCs with treatment of Control, radiation combined with or without anti-GRP78 pretreatment.

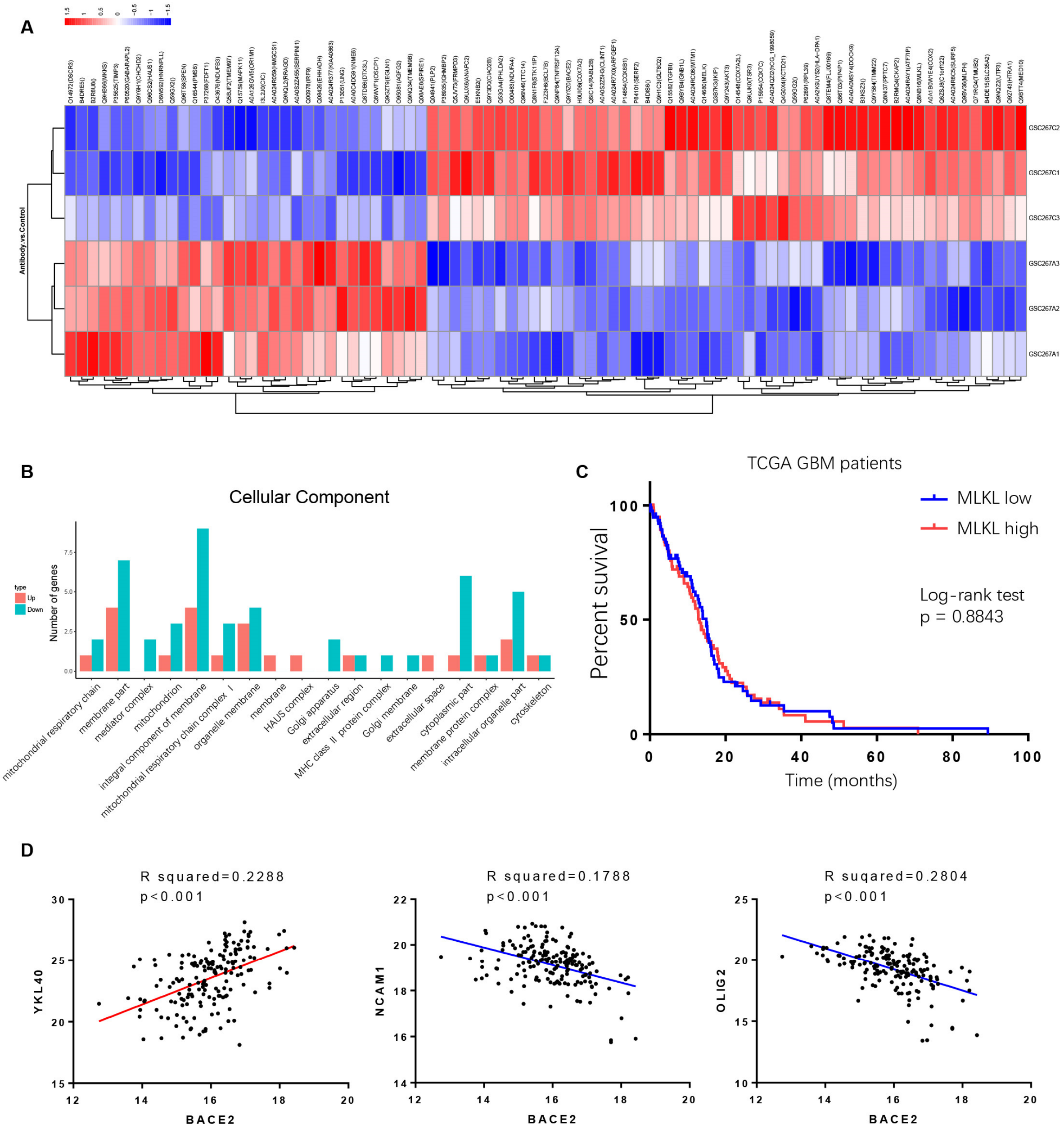

Supplementary figure 4

A. Heat map with detailed gene name of differential proteins in GSC267 treated with anti-GRP78 for 72 h.

B. Gene Ontology analysis for differential genes in MS revealed number of genes contributing to cellular components.

C. Survival curve and statistical analysis for GBM patients from TCGA based on MLKL mRNA expression.

D. Correlation of BACE2 expression with YKL40, NCAM1 or OLIG2 in TCGA database for glioma.

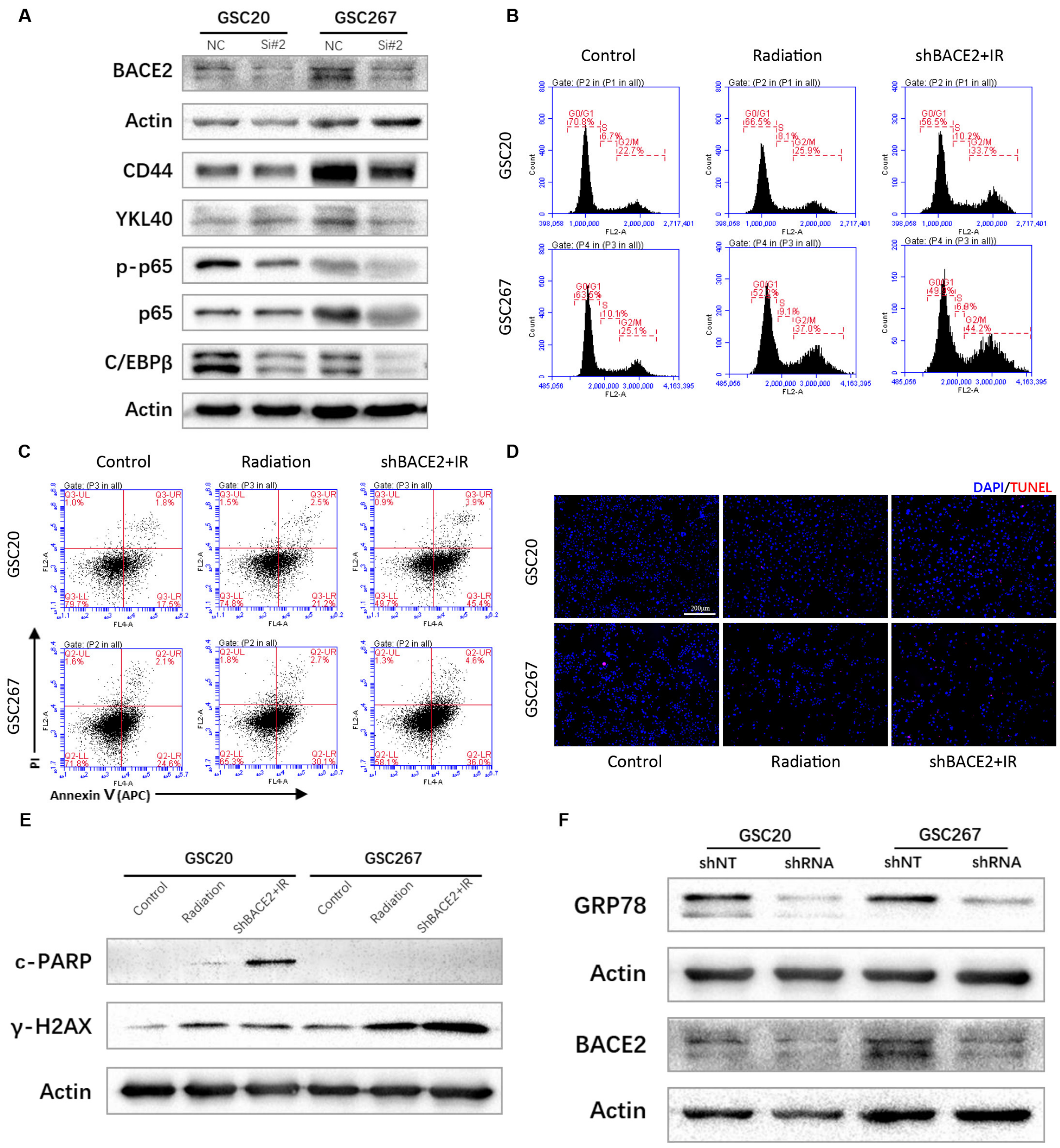

Supplementary figure 5

A. Same blot as Figure 5A, another effective siBACE2 sequence targeted total BACE2 in GSC20 and 267.

B. C6 flow cytometer plots of Figure 5D.

C. C6 flow cytometer plots of Figure 5E.

D. TUNEL staining of GSC20 and 267 with treatment of control, radiation only and radiation combined with shBACE2. Scale bar, 200 μm.

E. Western blotting for cleaved-PARP and γ-H2AX in GSCs grouped as designed.

F. Western blotting for GRP78 and BACE2 in GSC20 and 267 that transfected with lentiviral shGRP78 or shBACE2.

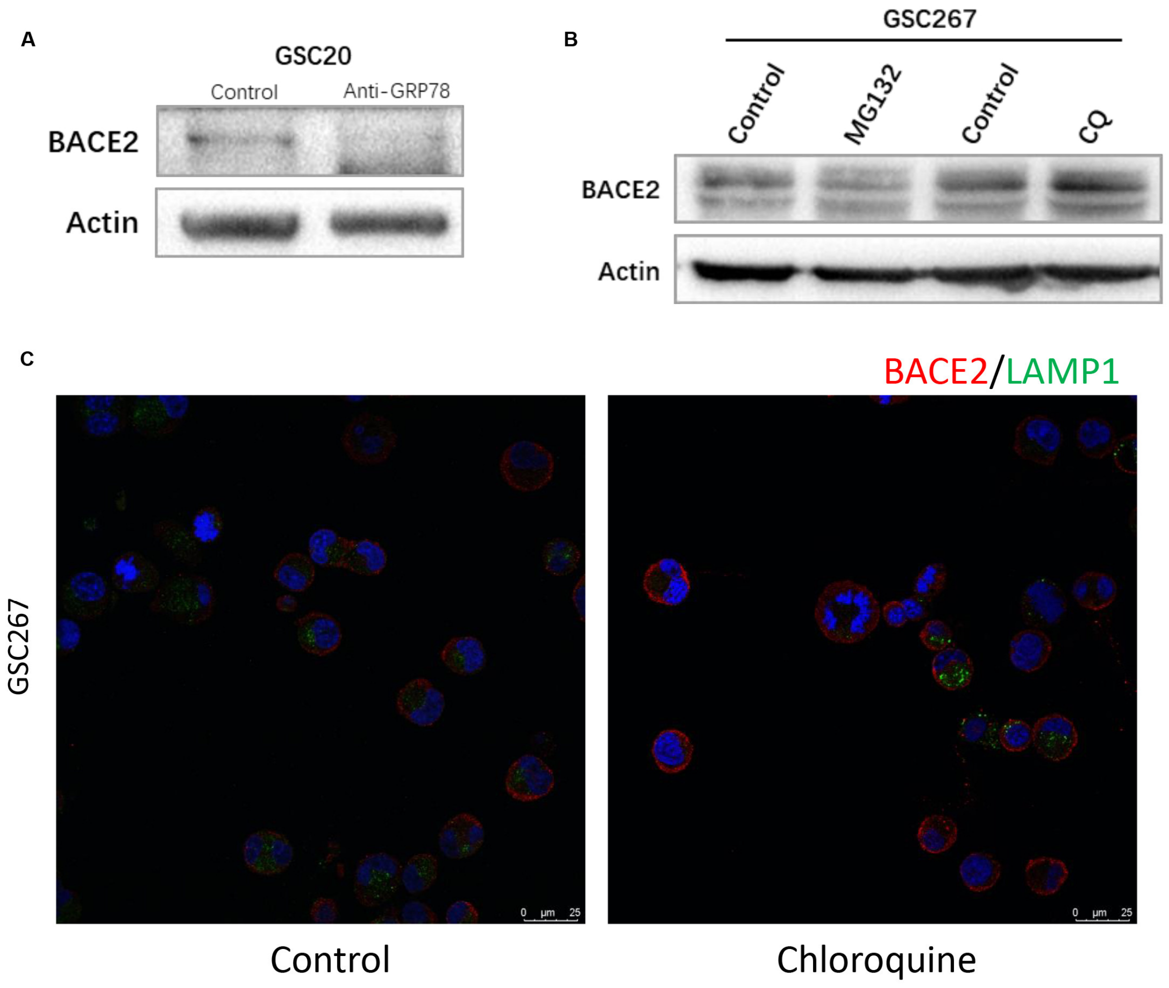

Supplementary figure 6

A. Western blotting of BACE2 in GSC20 treated with anti-GRP78 for 72 h.

B. Western blotting for BACE2 in GSC267 treated with MG-132 or chloroquine (CQ) for indicated time.

C. Immunofluorescence for BACE2 (red) and LAMP1 (green) in GSC267 treated with chloroquine for indicated time. Scale bar, 25 μm.

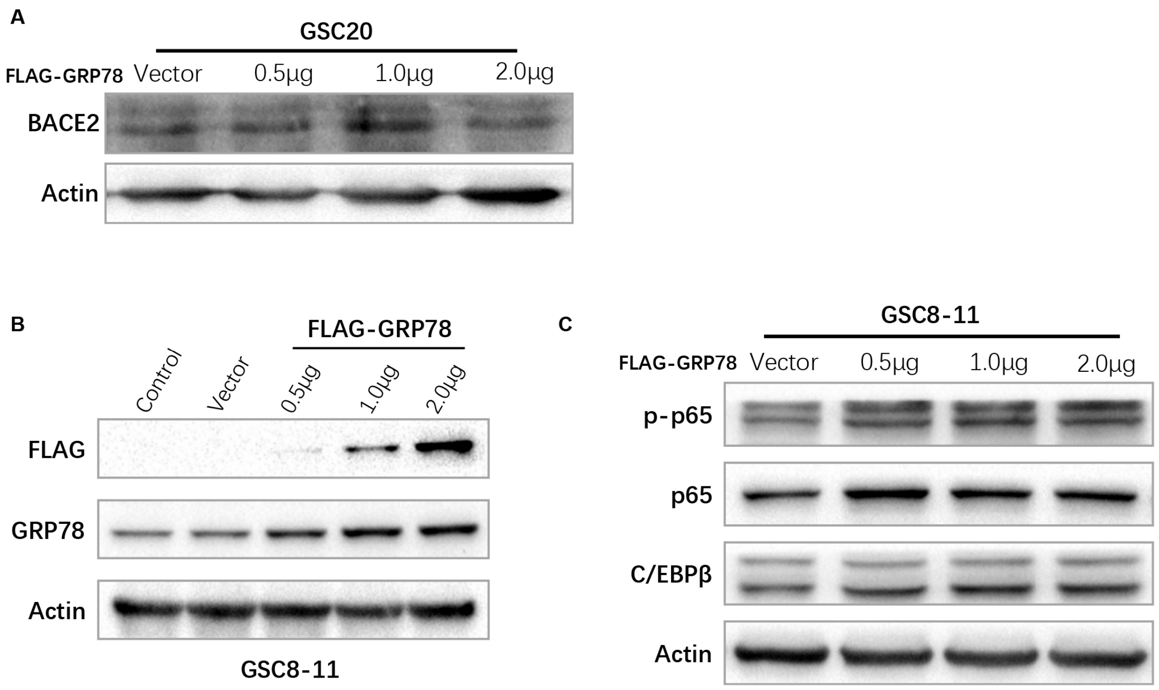

Supplementary figure 7

A. Western blotting of BACE2 in GSC20 transfected with WT FLAG-GRP78 or vector for 48 h.

B. Western blotting for FLAG tag and total GRP78 in GSC8-11 transfected with WT FLAG-GRP78 or vector for 48 h, comparing with control.

C. Western blotting for p-p65, p65 and C/EBPβ in GSC8-11 transfected with WT FLAG-GRP78 or vector.
